# Pediatric Autoimmune Encephalitis: Case Series From Two Chinese Tertiary Pediatric Neurology Centers

**DOI:** 10.3389/fneur.2019.00906

**Published:** 2019-08-22

**Authors:** Jianzhao Zhang, Taoyun Ji, Qian Chen, Yanan Jiang, Huan Cheng, Ping Zheng, Wenqiang Ma, Ting Lei, Yao Zhang, Yiwen Jin, Cuijie Wei, Ye Wu, Xingzhi Chang, Xinhua Bao, Yuehua Zhang, Hui Xiong, Xinna Ji, Shuo Feng, Haitao Ren, Jian Yang, Yuwu Jiang

**Affiliations:** ^1^Division of Pediatric Neurology, Pediatrics Department, Peking University First Hospital, Beijing, China; ^2^Department of Pediatric Neurology, Children's Hospital Affiliated to the Capital Institute of Pediatrics, Beijing, China; ^3^Department of Neurology, Peking Union Medical College Hospital, Chinese Academy of Medical Science, Beijing, China

**Keywords:** NMDAR, autoimmune encephalitis, child, prognosis, MOG

## Abstract

**Background and purpose:** We retrospectively analyzed the clinical characteristics of children with autoimmune encephalitis (AE) in two Chinese tertiary pediatric neurology centers. We also compared anti-NMDAR encephalitis with and without co-positive MOG antibody, as well as specific autoantibody-positive AE and autoantibody-negative but probable AE.

**Methods:** A retrospective study of children (0–18 years old) with AE in Peking University First Hospital and Children's Hospital Affiliated to Capital Institute of Pediatrics was carried out from May 2012 to January 2017. Demographics, clinical features, laboratory, and imaging findings, outcome, and co-positivity with MOG antibody were analyzed.

**Results:** A total of 103 children had AE, 89 (86.4%) had anti-NMDAR encephalitis, 2 (1.9%) had anti-LGI1 encephalitis, 1 (0.9%) had anti-CASPR2 encephalitis, and 11 (10.7%) were diagnosed as autoantibody-negative but probable AE. Among the 89 children with anti-NMDAR encephalitis, 35 were males and 54 were females. The follow-up time was 1–3 years. A total of 15 cases (15/89, 16.9%) with anti-NMDAR encephalitis had co-positive MOG antibody (serum or cerebrospinal fluid or both). These patients were more likely to experience relapse later in life (*P* = 0.014). We had two cases with anti-LGI1 encephalitis, that is, one with sleep disorder onset, and the other one with seizure onset, both of whom recovered after treatment. One case with anti-CASPR2 encephalitis was treated with an antiepileptic drug and fully recovered. There were 11 cases diagnosed as autoantibody-negative but probable AE who had relatively poorer outcome than those with autoantibody-positive AE (15.2%, 14/89). However, the difference was not significant (*P* = 0.08). Only one 12-year-old girl with NMDAR-antibody AE had ovarian teratoma.

**Conclusion:** Most subjects with AE in our Chinese cohort had anti-NMDAR AE, which had relatively good prognosis. Children with anti-LGI1 or anti-CASPR2 encephalitis were rare and showed good response on immunotherapy. Co-positive MOG antibody was relatively common in anti-NMDAR encephalitis, which was related to high relapse rate. In our study, the prognosis of autoantibody-negative but probable AE seemed worse than that of specific autoantibody-positive AE.

## Introduction

Autoimmune encephalitis (AE) is a brain disease caused by antibodies targeting neurons in the central nervous system to generate specific immune responses. Although immune encephalitis can occur at all ages, children's AE has unique characteristics. AE associated with cell surface antigens is more common in children, the most common of which is anti-NMDAR encephalitis, and other types of AE, such as LGI1 antibody-related AE, have also been reported (1–3). The common clinical manifestations of AE include abnormal mental behavior, seizure, abnormal memory and cognitive function, and motor and consciousness disorders. Cerebrospinal fluid and serum antibody detection is crucial to determine the specific type of AE. However, some patients were diagnosed with AE clinically but were autoantibody negative. In 2016, the new diagnostic criteria about autoantibody-negative but probable AE was established (4). The immunotherapy should be given as early as possible for AE. Although there is much in the literature about AE (5–7), regarding specifically for children's AE it is still limited. Therefore, we analyze the clinical characteristics, treatment, and prognosis of children with AE in two Chinese tertiary pediatric neurology centers herein.

## Subjects and Methods

### Subjects

The study was approved by the Ethics Committee of the Peking University First Hospital.

The data of children with AE who were hospitalized from May 2012 to January 2017 in the of Peking University First Hospital and children's hospital affiliated to the Capital Institute of Pediatrics were collected.

### Methods

**Figure 1:**
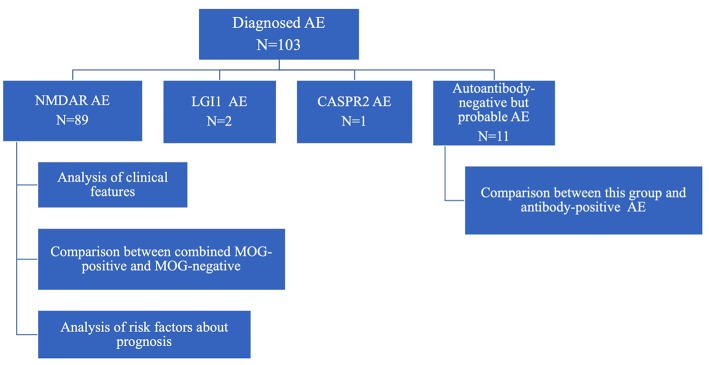
Flow diagram of study.

The diagnostic criteria for autoantibody-negative but probable AE and definite antibody encephalitis was proposed by Graus et al. (4) in 2016. AE was diagnosed by pediatric neurologists in each hospital on the basis of clinical findings and the presence of specific antibodies in CSF. The flow diagram of this study is shown in Figure 1.

The serum and CSF samples of each patient were sent to Oumeng Biotechnology Corporation, Beijing, China, or Neurological Lab, Peking University First Hospital, China, for the antibodies against the NMDA receptor and other AE-related antibodies. All samples were analyzed by indirect immunofluorescence assay using the EU 90 cells transfected method (BIOCHIPs, Euroimmun AG, Lubek, Germany).

We summarized the symptoms, such as psychiatric symptoms, seizures, speech disturbance, sleep disturbance, dyskinesia, and movement disorders, consciousness disturbance, memory deficit, and autonomic instability. Clinical data including age, gender, symptoms, CSF analysis, brain magnetic resonance imaging (MRI), electroencephalography (EEG), treatment, and follow-up were reviewed. First-line immunotherapy included intravenous (IV) methylprednisolone or intravenous immunoglobulins (IVIG), or a combination of these. Rituximab or cyclophosphamide treatment was defined as second-line immunotherapy.

All patients were followed for at least 1 year (in the range of 1–5). Epilepsy was diagnosed when seizure lasted for more than 24 months after the encephalitis (post-encephalitis epilepsy). Good outcome was defined as no sequela, and poor outcome as having any sequela.

### Statistical Analysis

Statistical analysis was conducted using SPSS 25.0. Data conformance to normal distribution is described by mean ± SE. Fisher's exact test was used to compare the categorical data. All predictors were tested in univariate models, the statistically significant indicators of the univariate analysis were added to the multivariate analysis, and the indicators considered probably to be clinically meaningful based on previous literature were also included in the multivariate analysis.

Associations were described as odds ratio used in developing the outcome in patients with each predictor relative to those without the predictor with 95% confidence interval and *P*-value. *P* < 0.05 was considered statistically significant.

## Results

### Clinical Demographics

**Figure 2:**
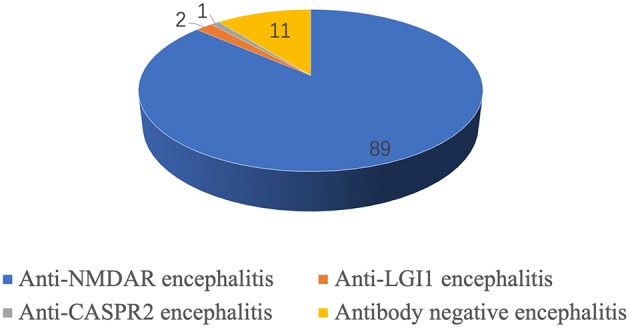
Autoimmune encephalitis classification in children.

A total of 103 children with AE, including 89 with anti-NMDAR encephalitis, two with anti-LGI1 encephalitis, one with anti-CASPR2 encephalitis, and 11 with autoantibody-negative but probable AE, were followed up (Figure 2).

### Characteristics of Children With Anti-NMDAR Encephalitis (Table 1)

The characteristics of anti-NMDAR encephalitis are as follows: 72 patients (80.9%) presented psychiatric symptoms, 65 (73.0%) experienced seizures, 65 (73.0%) had movement disorders, 60 (67.4%) had language disorders, 57 (64.0%) had memory disorders, and 43 (48.3%) had sleep disorders, followed by consciousness disturbance, paralysis, ataxia, sensory disturbance, and central hypoventilation. All patients underwent cranial MRI. Radiologists reported that 29 patients (32.6%) were abnormal. The abnormal locations of cranial MRI in 21 (23.6%), 7 (7.9%), 7 (7.9%), and 5 patients (5.6%) were found in the temporal lobe, frontal lobe, parietal lobe, and basal ganglia, respectively. EEG was performed in all patients, and 79 patients (88.8%) obtained abnormal findings; 42 patients (47.2%) had generalized slow-wave, 33 (37.1%) had focal slow-wave, 55 (61.8%) had epileptic discharge, and 15 patients (16.8%) exhibited extreme delta brush. The CSF of all patients was positive for NMDAR-IgG, but 60 patients (67.4%) had positive NMDAR-IgG in serum. A total 41 patients (46.1%) had CSF leukocytosis (>5/mm^3^). A total of 52 patients (58.4%) had oligoclonal band positive in CSF. MOG-positive serum or CSF was found in 15 patients (16.9%). For treatment, glucocorticoid therapy was performed in 87 patients (97.8%), intravenous immunoglobulin (IVIG) treatment was performed in 77 patients (86.5%), second-line drugs (rituximab and cyclophosphamide) were used in 32 patients (35.9%), and two children (2.2%) did not use immunotherapy because their parents refused to use it. Prognosis showed that 75 patients (84.3%) had complete recovery, six patients (6.7%) had epilepsy, six (6.7%) had cognitive dysfunction, one (1.1%) exhibited ataxia, and one (1.1%) died. A total of 12 patients (13.5%) experienced relapse.

**Table 1:** Clinical characteristics of children with anti-NMDAR encephalitis.

**Demographic and clinical characteristics**	**0–3 years old**	**3–6 years old**	**6–12 years old**	**12–18 years old**	**Total**
Total	11	24	39	15	89
Seizures Seizures as initial symptom	8 (72.7%) 6 (54.5%)	20(83.3%) 15(62.5%)	27 (69.2%) 13 (33.3%)	10 (66.7%) 7 (46.7%)	65(73.0%) 41(46.1%)
Psychiatric symptom Psychiatric as initial symptom	8 (72.7%) 3 (27.3%)	21(87.5%) 8 (33.3%)	31 (79.5%) 14 (35.9%)	12 (80.0%) 6 (40.0%)	72(80.9%) 31(34.8%)
Movement disorders Movement disorder as initial symptom	7 (63.6%) 1 (9.1%)	20(83.3%) 2 (8.3%)	27 (69.2%) 4 (10.3%)	11 (73.3%) 2 (13.3%)	65(73.0%) 9 (10.1%)
Speech dysfunction Speech dysfunction as initial symptom	3 (27.3%) 0	17(70.8) 2 (8.3%)	30 (76.9%) 5 (12.8%)	10 (66.7%) 2 (13.3%)	60(67.4%) 9 (10.1%)
Sleep disorder	4 (36.4%)	14(58.3)	18 (46.2%)	7 (46.7%)	43(48.3%)
Memory disorder	5 (45.5%)	13(54.2%)	28 (71.8%)	11 (73.3%)	57(64.0%)
Consciousness disturbance	5 (45.5%)	7 (29.2%)	11 (28.2%)	6 (40.0%)	29(32.6%)
Ataxia	1 (9.1%)	4 (16.7%)	8 (20.5%)	2 (13.3%)	15(16.9%)
Sensory disorder	0	0	4 (10.3%)	0	4 (4.5%)
Paralysis	6 (54.5%)	5 (20.8%)	5 (12.8%)	1 (6.7%)	17(19.1%)
Hypoventilation	0	0	2 (5.1%)	0	2 (2.2%)
Cranial MRI with abnormal findings			29 (32.6%)		
Temporal lobe	1 (9.1%)	6 (25.0%)	12 (30.8%)	2 (13.3%)	21(23.6%)
Frontal lobe	1 (9.1%)	2 (8.3%)	4 (10.3%)	0	7 (7.9%)
Parietal lobe	0	2 (8.3%)	5 (12.8%)	0	7 (7.9%)
Basal ganglia	1 (9.1%)	2 (8.3%)	2 (5.1%)	0	5 (5.6%)
Brain stem	0	1 (4.2%)	2 (5.1%)	1 (6.7%)	4 (4.5%)
Cerebellum	0	1 (4.2%)	1 (2.6%)	1 (6.7%)	3 (3.4%)
Thalamus	0	0	1 (2.6%)	1 (6.7%)	2 (2.2%)
Occipital lobe	0	0	2 (5.1%)	0	2 (2.2%)
Deep white matter	2 (18.2%)	4 (16.7%)	1 (2.6%)	1 (6.7%)	8 (9.0%)
Subcortical white matter	1 (9.1%)	5 (20.8%)	3 (7.7%)	1 (6.7%)	10(11.2%)
EEG with abnormal findings			79 (88.8%)		
Focal slowing	2 (18.2%)	8 (33.3%)	16 (41.0%)	7 (46.7%)	33(37.1%)
Generalized slowing	6 (54.5%)	12(50.0%)	18 (46.2%)	6 (40.0%)	42(47.2%)
Epileptic form discharge	7 (63.6%)	16(66.7%)	24 (61.5%)	8 (53.3%)	55(61.8%)
Extreme delta brush	4 (36.4%)	3 (12.5%)	5 (12.8%)	3 (20.0%)	15(16.9%)
CSF pleocytosis (>5/mm^3^)	2 (18.2%)	11(45.8%)	21 (53.8%)	7 (46.7%)	41(46.1%)
CSF Oligoclonal band	8 (72.7%)	13(54.2%)	24 (61.5%)	7 (46.7%)	52(58.4%)
MOG-positive (serum or CSF)	2 (18.2%)	5 (20.8%)	7 (17.9%)	1 (6.7%)	15(16.9%)
Immunotherapy					
Steroid only	10(90.9)	24(100%)	39 (100.0%)	14 (93.3%)	87(97.8%)
IVIG only	8 (72.7%)	20(83.3%)	35 (89.7%)	14 (93.3%)	77(86.5%)
Second-line drugs (rituximab or cyclophosphamide)	5 (45.5%)	11(45.8%)	14 (35.9%)	2 (13.3%)	32(35.9%)
No immunotherapy	1 (9.1%)	0	0	1 (6.7%)	2 (2.2%)
Relapse	1 (9.1%)	2 (8.3%)	7 (17.9%)	2 (13.3%)	12(13.5%)
Prognosis					
Complete recovery	10(90.9%)	17(70.8%)	35 (89.7%)	13 (86.7%)	75(84.3%)
Epilepsy	0	4 (16.7%)	0	2 (13.3%)	6 (6.7%)
Cognitive dysfunction	1 (9.1%)	2 (8.3%)	3 (7.7%)	0	6 (6.7%)
Ataxia	0	0	1 (2.6%)	0	1 (1.1%)
Death	0	1 (4.2%)	0	0	1 (1.1%)

### Analysis of Factors Regarding Anti-NMDAR Encephalitis Outcome (Tables 2, 3)

The results of univariate analysis are shown in Table 2. On multivariate regression analysis, the factors associated with anti-NMDAR encephalitis outcome were admission to ICU (*P* = 0.016) and status epilepticus (*P* = 0.023, Table 3).

**Table 2:** Factors associated with outcome of anti-NMDAR encephalitis: univariate analysis.

	**Complete recovery**	**Incomplete recovery**	**OR (95% CI) or *t*-value**	***P*-value**
Potential predictors	75	14		
Age (mean ± SE, year)	7.92 ± 3.89	6.85 ± 4.96	−1.112	0.664
Female (%)	45 (60.0%)	9 (64.3%)	0.881 (0.269–2.891)	0.835
Status epilepticus	22 (29.3%)	9 (64.3%)	4.336 (1.305–14.411)	0.017
Consciousness disturbance	22 (29.3%)	7 (50.0%)	4.07 (1.228–13.489)	0.022
Movement disorder	52 (69.3%)	13 (92.9%)	5.396 (0.665–43.795)	0.115
CSF pleocytosis (≧5/mm^3^)	34 (45.3%)	7 (50.0%)	1.072 (0.327–3.522)	0.908
Anti-NMDAR body titer ≧100	30 (40.0%)	10 (71.4%)	3.75 (1.076–13.065)	0.038
Abnormal cranial MRI	23 (30.7%)	6 (42.8%)	1.594 (0.497–5.106)	0.433
EEG with abnormal findings (slow wave or epileptic form discharge)	66 (88.0%)	13 (92.8%)	1.773 (0.207–15.217)	0.602
Extreme delta brush	12 (16.0%)	3 (21.4%)	1.97 (0.460–8.435)	0.361
ICU admission	3 (4.0%)	4 (28.6%)	9.6 (1.869)	0.007

**Table 3:** Factors associated with anti-NMDAR encephalitis outcome: multivariate analysis.

	**Odds ratio**	**95% CI**	***P*-value**
Status epilepticus	5.329	1.26–22.529	0.023
Consciousness disturbance	1.235	0.319–22.529	0.760
Movement disorder	2.944	0.302–28.696	0.353
Abnormal cranial MRI	1.455	0.331–6.388	0.619
Abnormal EEG	2.113	0.177–25.219	0.554
Anti-NMDAR antibody titer ≧ 100	1.821	0.447–7.415	0.403
ICU admission	11.494	1.569–84.2	0.016

### Comparison Between Combined MOG Antibody-Positive and -Negative Children With Anti-NMDAR Encephalitis (Table 4)

A higher proportion of precursor infection and relapse was found in MOG antibody-positive children than those in MOG antibody-negative ones, and the difference was statistically significant (*P* < 0.05).

**Table 4:** Comparison between combined MOG antibody-positive and -negative children with anti-NMDAR encephalitis.

	**MOG-positive**	**MOG-negative**	***X^**2**^* or *t*-value**	***P*-value**
Total	15	74		
Age (mean ± SE, year)	6.31 ± 3.82	7.60 ± 3.92	−1.162	0.248
Preceding infection	8 (53.3%)	18 (24.3%)	5.075	0.024
Status epilepticus	5 (33.3%)	26 (35.1%)	1.103	0.218
Consciousness disturbance	6 (40.0%)	23 (31.1%)	0.452	0.502
CSF pleocytosis (≧5/mm^3^)	7 (46.7%)	34 (43.2%)	0.002	0.964
Anti-NMDAR antibody titer ≧100	7 (46.7%)	33 (44.6%)	0.003	0.959
CSF Oligoclonal band	9 (60.0%)	43 (58.1%)	0.018	0.892
Cranial MRI with abnormal white matter	4 (26.7%)	14 (18.9%)	0.464	0.496
Extreme delta brush	1 (6.7%)	14 (18.9%)	1.336	0.248
ICU admission	1 (6.7%)	6 (8.1%)	0.036	0.850
Relapse	5 (33.3%)	7 (9.5%)	6.094	0.014
Second-line immunology	6 (40.0%)	26 (35.1%)	0.066	0.797
Sequela	2 (13.3%)	12 (16.2%)	0.078	0.780

### Clinical Analysis of Children With Anti-LGI1 Encephalitis (Table 5)

Two patients had anti-LGI1 encephalitis, one of which was an 8-year-old boy with clinical manifestation mainly for insomnia. The cranial MRI of this patient showed left hippocampal lesions and showed positive CSF and serum LGI1 antibody. Without ICU admission, video EEG showed focal slow waves. The number of cerebrospinal fluid cells was normal. After 2 weeks of treatment with IVIG, the clinical manifestations and cranial MRI significantly improved. The second patient was a 15-year-old boy with seizure. Anti-LGI1-IgG antibody was positive (1:100) in the serum. No memory loss, cognitive impairment, mental disorder, sleep disorder, or movement disorders were reported. The prognosis was good by using IVIG (2 g/kg, for 5 days) and levetiracetam for 1 year.

**Table 5:** Clinical characteristics of anti-LGI1 and anti-CASPR2 AE.

**Patients**	**Anti-LGI1 patient 1**	**Anti-LGI1 patient 2**	**Anti-CASPR2**
Age	8 years old	15 years old	5 years old
Gender	Male	Male	Male
Seizures	No	Yes	Yes
Status epilepticus	No	No	No
Psychiatric symptom	No	No	No
Movement disorders	No	No	No
Speech dysfunction	No	No	No
Sleep disorder	Yes	No	No
Memory disorder	No	No	No
Ataxia	No	No	No
Paralysis	No	No	No
Hypoventilation	No	No	No
Cranial MRI with abnormal findings	Yes	No	No
EEG with abnormal findings	Yes (focal slow waves)	Yes (focal slow waves and Epileptic form discharge)	Yes (focal slow waves)
CSF pleocytosis (>5/mm^3^)	No	No	No
CSF Oligoclonal band	No	No	No
MOG-positive (serum or CSF)	No	No	No
Immunotherapy			
Steroid	No	No	No
IVIG	Yes	Yes	No
Second-line drugs (rituximab or cyclophosphamide)	No	No	No
Anti-epilepsy drugs	No	Yes	Yes
ICU admission	No	No	No
Complete recovery	Yes	Yes	Yes

### Anti-CASPR 2 Encephalitis (Table 5)

One of the children was a 5-year-old boy who was admitted to the hospital for 1 day due to paroxysmal headache and vomiting for 2 months was diagnosed with anti-CASPR 2 encephalitis. During the course of the disease, convulsions lasted for 1 h and 30 min. Cranial MRI showed no abnormality, and EEG indicated slow waves in the occipital region. CSF test was normal, and serum anti-CASPR 2-IgG was positive. There was no ICU admission. No convulsions were observed for more than 2 years after the levetiracetam treatment, and the cognitive function of this patient was normal. Parents refused immunotherapy for this child.

### Analysis of Autoantibody-Negative but Probable AE

A total of 11 patients were diagnosed with autoantibody-negative but probable AE. All patients were followed up for 1–2 years. Six patients were female, and their mean age was 6.18 ± 2.09 years old. Seizures were observed in all patients, mental symptoms were found in nine patients, and dyskinesia was presented in two patients. EEG showed generalized or focal slow-wave in all patients. Five patients exhibited epilepsy discharge, and all patients had cranial MRI abnormalities. Two patients did not receive immunotherapy. In terms of prognosis, two patients experienced epilepsy, one patient had dyskinesia, and one patient exhibited irritability. In this group, seven patients were cured, and four had sequelae.

### Comparison Between Children With Autoantibody-Negative but Probable AE and With Antibody-Positive AE (Table 6)

In contrast to the antibody-positive encephalitis group, the proportions of movement disorders and CSF oligoclonal band were higher than those of the antibody-negative group (*P* < 0.05). The number of cluster seizures in the autoantibody-negative but probable AE group was higher than that in the antibody-positive encephalitis group (*P* < 0.05).

**Table 6:** Comparison between children with autoantibody-negative but probable AE and antibody-positive AE.

	**Negative**	**Positive**	***X^**2**^* or *t*-value**	***P*-value**
Total	11	92		
Age (mean ± SE year)	6.20 ± 2.26	7.29 ± 2.87	0.017	0.334
Female (%)	6 (54.5%)	54 (58.7%)	0.227	0.634
Psychiatric symptom	8 (72.7%)	72 (78.3%)	0.109	0.742
Movement disorder	4 (36.3%)	65 (70.7%)	4.828	0.028
Cluster seizures	9 (81.8%)	42 (45.7%)	5.141	0.023
Status epilepticus	4 (36.4%)	31 (33.7%)	0.063	0.802
Consciousness disturbance	4 (36.4%)	29 (31.5%)	0.106	0.745
CSF pleocytosis(≧5/mm^3^)	5 (45.5%)	41(44.6%)	0.003	0.955
CSF Oligoclonal band	2 (18.2%)	52 (56.5%)	5.791	0.016
ICU admission	1 (9.1%)	7 (7.6%)	0.03	0.862
Relapse	1 (9.1%)	12 (13.0%)	0.057	0.746
Second-line immunology	3 (27.3%)	32 (34.8%)	0.247	0.619
Sequelae	4 (36.4%)	14 (15.2%)	3.046	0.081

### AE in Children With Tumor

All the children underwent chest- and abdomen-enhanced CT examination, and the boys underwent testicular ultrasound examination. Only one 12-year-old girl with anti-NMDAR encephalitis had ovarian teratoma (0.9%, 1/103). No tumors were found in children younger than 12 years old, and no patient with other AE had a tumor.

## Discussion

With the discovery of relevant antibodies, the etiology of some unknown causes of encephalitis has been clarified, and AE has become a topic receiving considerable interest in research. However, most of the published works on AE focus upon adult patients. Studies on children with AE are relatively few, or the analysis was not specific enough (5–8). Therefore, we analyze the cases of AE (including AE with known and unknown antibodies) in two Chinese tertiary pediatric neurology centers, of which both hospitals had patients from all over the country, thereby representing Chinese children with AE to some extent.

The most common clinical features of anti-NMDAR AE as the initial symptoms in our study were seizures, psychiatric symptoms, language disorders, movement disorders, and sleep disorders. Seizure is also the most common symptom in children, which is consistent with much literature (4, 5, 9–11). Children always manifested with neurological symptoms onset, adults with psychiatric symptoms (11). In the children's anti-NMDAR AE, the onset of epilepsy as the initial symptom reached 72%, and the form of epileptic attack was the most common (58%) and comprehensive attack (42%) (11). In adult patients, only 14% (12, 13) of patients are onset of seizures presented as the initial symptom. Other symptoms, such as psychiatric symptoms, involuntary movements, language disorders, and sleep disturbances, are as common as reported in other literature.

The predictors of poor outcome were status epilepticus and ICU admission. In previous studies, the predictors of poor outcome included delayed treatment, young age, decreased consciousness, memory deficiency, high antibody titers, and ICU admission (11, 14, 15). ICU admission was a predictor of poor outcome, which was consistent with our study, whereas the status epilepticus as a predictor of poor outcome in our study is different from previous literature.

The concomitancy of anti-NMDAR antibody and MOG antibody has been reported recently (16, 17). In our cases, one patient was diagnosed with acute disseminated encephalomyelitis due to acute multiple demyelinating disease, and the test showed that the patient was NMDAR antibody-positive. Thus, we should pay attention in identifying demyelinating or acute demyelinating diseases combined with anti-NMDAR encephalitis. In the study of Titulaer et al. (18), the cohorts were divided into three groups. Group 1 included 12 patients whose anti-NMDAR encephalitis was preceded or followed by independent neuromyelitis or demyelinating syndromes (seven cases, all anti-MOG antibody-positive). Group 2 included 11 patients whose anti-NMDAR encephalitis occurred with MRI abnormality and symptoms compatible with demyelination (two MOG antibody-positive cases). Group 3 included 50 randomly selected patients with typical anti-NMDAR encephalitis (three MOG antibody-positive cases). In our cohort, MOG antibody-positive serum or CSF in 15 (16.9%) patients in anti-NMDAR encephalitis was higher than those in other reports. The reason may be that measuring positive for MOG-antibody is considerably high in children. The incidence of MOG-Ab often occurs in East Asia (19). Our patients also had increased risk of relapse later in life (*P* = 0.014) and a high proportion of preceding infection (*P* = 0.024). MOG is a specific glycoprotein in the white matter of the central nervous system. Anti-MOG antibodies can cause demyelinating lesions. In this study, 18 patients had MRI demyelinating lesions, of which 4 were positive for MOG antibodies, but 14 patients did not find any demyelinating related antibodies, including MOG and AQP4. In addition, there were 11 patients with positive MOG antibodies, but no demyelinating lesions were found on MRI. The mechanism by which MOG antibodies and NMDA antibodies are simultaneously positive is still unknown.

A previous study Dalmau et al. (9) reported that 55% of those with anti-NMDAR encephalitis had abnormal cranial MRI, and the lesions were located in the temporal lobe, hippocampus, corpus callosum, cerebellum/cerebellum cortex, basal ganglia, and brainstem. A multicenter study Schimmel et al. (1) of 540 patients with anti-NMDAR encephalitis showed that 33% of the patients had cranial MRI abnormalities, and 80% of the abnormal signals were found in the temporal and frontal lobes. A total of 29 cases (32.6%) of children with cranial MRI abnormalities located in the temporal, frontal, and parietal lobes were reported in our study. The high proportion of basal ganglia, the incidence of cranial MRI abnormalities, and lesions in the study of this area were consistent with the results in the literature, but the pathological feature and specificity of the lesion site are lacking. A total of 79 out of the 89 patients had abnormal EEGs (88.7%), which were mainly composed of diffused slow-wave, followed by focal slow-wave. However, the extreme delta brush was rare. This finding was also reported in some previous studies (6, 20).

Autoimmune encephalitis (AE) therapy mainly includes first-line and the second-line immunotherapy. A previous work Zekeridou et al. (12) and this study showed that glucocorticoid is still the most frequently used first-line drug. Second-line drugs are always used in children with severe illness or relapse, around 20–30% of total patients. Most of the children with anti-NMDAR encephalitis had relatively good prognosis. Seventy-five patients (84.3%) achieved good outcomes, while 15 patients had poor outcomes in our study. The ratio of good outcomes was lower than those in previous research because we considered cognitive impairment an indicator of poor outcome, which was less used in previous studies. Only one (1.1%) patient died in our study, which was similar to that in previous studies, that is, the death rate in young children is low (2.7%) (11). This finding may be associated with the low proportion of cancer and autonomic instability. Twelve patients (13.5%) relapsed and improved after second-line treatment, which was consistent with the results of a previous report (12%) (11). Relapse rate can also reach 20–24% (21, 22), but the patients in those studies were adults only or both adults and children.

Our study found two cases with anti-LGI1 encephalitis and one case with anti-CASPR2 encephalitis. For two cases with LGI1 encephalitis, their first symptom was only sleep disturbance or seizure, their cranial MRI had typical characteristics, and they both responded well to immunotherapy. Only one case with anti-CASPR2 encephalitis was found, which was mainly manifested as consciousness disturbance and seizure. The seizure was controlled after antiepileptic treatment.

Some patients can be diagnosed with AE in clinical manifestation without specific antibodies. According to the proposed diagnosis criteria (4) for autoantibody-negative but probable AE, we diagnosed 11 patients [10.7% (11/103)], which was higher than that reported previously (7%) (23). These patients were given immunotherapy and were observed for 1–2 years. The outcomes showed that two cases had epilepsy, one had dyskinesia, and one had a sharp temper. The comparison between antibody-positive and -negative encephalitis groups showed that the proportion of dyskinesia and CSF oligoclonal band was higher than those of the autoantibody-negative but probable AE group (*P* < 0.05). The cluster seizures in the autoantibody-negative but probable AE group were more frequent than in the antibody-positive encephalitis group (*P* < 0.05), which were not reported in the previous study. Compared with the other AE types in this study, the prognosis of patients with autoantibody-negative but probable AE was poor. Regarding the pathogenesis of antibody-negative encephalitis, some antibodies may have not yet been discovered. However, these patients may not be associated with autoantibody but related to abnormal cellular or innate immune process (24).

In all children with AE in our study, only one 12-year-old girl had ovarian teratoma in anti-NMDAR encephalitis, thereby suggesting that children were less likely to develop tumors than older people, which was consistent with the results of a previous report on a multicenter study (9) showing that the incidence of teratoma in patients with an age of >18 years old is 56%, thereby accounting for 31% of women with the age of <18 years old and 9% in women with the age of <14 years old. In our study, we only had one case of teratoma (1.1%). Thus, in the children with AE, the incidence of tumors is low, especially for young children. Therefore, according to the characteristics of childhood illness, infectious factors may be a major inducing factor in children.

In conclusion, AE in children has its own characteristics regardless of the first sign of the disease or the condition of tumor concomitant. The shortcoming of this study is that it is not a prospective study and does not use mRS to evaluate the function. Additional research, especially prospective studies to clarify the diagnosis and treatment of anti-NMDAR encephalitis in some subgroup of children, such as the treatment of anti-NMDAR encephalitis-related epileptic seizures, is still needed in the future.

## Ethics Statement

Ethics approval for this study was obtained from the Ethics Committee of the Peking University First Hospital. The parents of the patients signed written informed consent and agreed with the participation of their children in this study and allowed the use of the relevant data and information for scientific research.

## Author Contributions

JZ and TJ contributed in preparing the draft manuscript of this article and prepared the text. PZ and XJ prepared the figure. YJia and JY were responsible for all supervision and are the guarantor of the article. HR was responsible for the detection of specimens. Other authors have been involved in the management of the children.

### Conflict of Interest Statement

The authors declare that the research was conducted in the absence of any commercial or financial relationships that could be construed as a potential conflict of interest.
